# Cortical Presynaptic Control of Dorsal Horn C–Afferents in the Rat

**DOI:** 10.1371/journal.pone.0069063

**Published:** 2013-07-30

**Authors:** Yunuen Moreno-López, Jimena Pérez-Sánchez, Guadalupe Martínez-Lorenzana, Miguel Condés-Lara, Gerardo Rojas-Piloni

**Affiliations:** Departamento de Neurobiología del Desarrollo y Neurofisiología, Instituto de Neurobiología, Universidad Nacional Autónoma de México, Campus UNAM-Juriquilla, Querétaro, México; Emory University, United States of America

## Abstract

Lamina 5 sensorimotor cortex pyramidal neurons project to the spinal cord, participating in the modulation of several modalities of information transmission. A well-studied mechanism by which the corticospinal projection modulates sensory information is primary afferent depolarization, which has been characterized in fast muscular and cutaneous, but not in slow-conducting nociceptive skin afferents. Here we investigated whether the inhibition of nociceptive sensory information, produced by activation of the sensorimotor cortex, involves a direct presynaptic modulation of C primary afferents. In anaesthetized male Wistar rats, we analyzed the effects of sensorimotor cortex activation on post tetanic potentiation (PTP) and the paired pulse ratio (PPR) of dorsal horn field potentials evoked by C–fiber stimulation in the sural (SU) and sciatic (SC) nerves. We also explored the time course of the excitability changes in nociceptive afferents produced by cortical stimulation. We observed that the development of PTP was completely blocked when C-fiber tetanic stimulation was paired with cortex stimulation. In addition, sensorimotor cortex activation by topical administration of bicuculline (BIC) produced a reduction in the amplitude of C–fiber responses, as well as an increase in the PPR. Furthermore, increases in the intraspinal excitability of slow-conducting fiber terminals, produced by sensorimotor cortex stimulation, were indicative of primary afferent depolarization. Topical administration of BIC in the spinal cord blocked the inhibition of C–fiber neuronal responses produced by cortical stimulation. Dorsal horn neurons responding to sensorimotor cortex stimulation also exhibited a peripheral receptive field and responded to stimulation of fast cutaneous myelinated fibers. Our results suggest that corticospinal inhibition of nociceptive responses is due in part to a modulation of the excitability of primary C–fibers by means of GABAergic inhibitory interneurons.

## Introduction

The cortico–spinal tract performs multiple functions, including motor control as well as the modulation of different types of sensory information at the spinal cord level, including nociception (for review see [1]). However, the modulation of nociceptive sensory information mediated by the corticospinal system has been poorly studied, and the mechanisms are not well understood. Existing studies in animal models have reported that the stimulation of sensorimotor cortices modulates the activity of nociceptive dorsal horn cells in monkeys [2], [3] and rats [4], [5], as well as synaptic C–fiber-evoked responses in the rat dorsal horn [6]. Additionally, clinical studies have shown that the stimulation of the motor cortex can be used successfully to manage chronic and drug-resistant peripheral neurophatic pain of various origins [7], [8], [9], [10], [11], [12], [13], [14], [15], [16], [17], [18].

Conversely, primary afferent depolarization (PAD), leading to presynaptic inhibition of sensory fibers and mediated by the cortical descending projection, has been documented since the 1960's [19], [20], [21], [22], [23], [24]. The cortico–spinal modulation of sensory inputs can be mediated directly by activation of segmental interneurons [22] or indirectly by extrapyramidal pathways [25]. In particular, sensorimotor cortex stimulation produces dorsal root potentials (DRP) and PAD in muscular and fast cutaneous afferents [22], [24], [26] without affecting group II afferents [27]. Presynaptic inhibition mediated by PAD has been proposed as one of the mechanisms underlying the modulation of nociceptive information mediated by the sensorimotor cortex. However, whether a cortical control is exerted on thinly myelinated and unmyelinated nociceptive fibers is still unknown.

The purpose of the present study was to evaluate if the inhibition of nociceptive sensory information, carried by C primary afferents and produced by activation of the sensorimotor cortex, involves presynaptic GABAergic control. To this end, we evaluated the effects of sensorimotor cortex activation on the post tetanic potentiation (PTP) and paired pulse ratio (PPR) of C–fiber evoked dorsal horn responses [28]. Furthermore, we tested the effect of sensorimotor cortex stimulation on the excitability of C–fiber terminal arborizations [28], [29]. We also analyzed the effects of bicuculline (BIC), applied to the surface of the spinal cord, on the inhibition of C–fiber neuronal responses mediated by cortical stimulation. Finally, we explored the location of dorsal horn neurons that are activated by the stimulation of the sensorimotor cortex.

## Methods

### Ethic statement

All procedures were carried out in strict accordance with the recommendations of the National Institutes of Health Guide for the Care and Use of Experimental Animals and were approved by the local Animal Research Committee of the Instituto de Neurobiología at Universidad Nacional Autónoma de México. 76 Wistar rats (280–310 g) were used for this study and all efforts were made to minimize the number of animals used and their suffering during the experiments. Additionally, in order to minimize trauma and the length of anesthesia, only one experimental protocol was performed per animal. The animals were housed individually in a temperature-controlled (24°C) colony room and maintained on a 12-h/12-h light/dark cycle (lights on at 7:00 A.M.). Food and water were provided ad libitum. At the end of the experiments the animals were euthanized by means of pentobarbital overdose (>120 mg/kg, i.v.).

### General procedures

The rats were anesthetized with urethane (1.4 grs/kg, i.p.), paralyzed with pancuronium (0.5 mg/kg, i.v.) and artificially respired. Tidal air volume was adjusted to have an end tidal CO_2_ concentration of 2–4%. The electrocardiogram was monitored during the experiment, and the core temperature was kept at 37°C by means of a heating pad. In some experiments, the left sciatic nerve (SN) was carefully exposed and isolated at the mid–thigh level. A pair of silver hook electrodes was placed around the SN for stimulation (Wire, Silver, Teflon coated, PHYMEP s.a.r.l. Paris). The exposed nerve was covered with mineral oil at 37°C. For the primary afferent excitability tests, the sural (SU) nerve was dissected free at the level of the popliteal fossa and cut at the periphery. The rats were fixed in a stereotaxic apparatus (Narishige SR-6R) and secured in a spinal cord unit frame (Narishige STS-B), thus fixing lumbar vertebrae to improve stability at the recording site. A laminectomy was performed to expose spinal cord segments L4–L6, and the surface of the exposed spinal cord was irrigated with saline solution.

### Recording and stimulation

Intraspinal potentials were recorded with the preamplifier filters set to a bandwidth of 0.3 Hz–10 kHz. During the experiment, all recordings were digitalized and stored for further processing. C-fiber and cortical evoked field potentials (EFPs) were recorded with glass micropipettes filled with 1.2 M NaCl (tip diameter, 1.0–2.5 µm; 1.2–1.7 MΩ). For the intraspinal recordings, micropipettes were placed at depths where negative C-fiber EFPs reached their maximal amplitude (300–400 µm; Lamina II–III). Spinal EFPs were produced by stimulation of the SU and SN with single pulses (0.1 ms), and the stimulation strength was adjusted to elicit a C-fiber response of 40 to 60% of the maximal amplitude (0.5–0.8 mA). In some experiments, intraspinal EFPs, DRPs, as well as single-unit extracellular recordings, produced by contralateral cortical stimulation were analyzed.

Cortical stimulation consisting of trains of 5 pulses (pulse duration 0.1 ms, 100 Hz, 100 µA) was carried out with bipolar stainless steel electrodes (0.5-mm separation between the tips) inserted 1000 µm below the cortical surface (around cortical layer 5). The stimulation electrodes were placed in the region where intraspinal EFPs evoked by cortical stimulation are maximal (3 mm from the midline and 2.5 mm posterior to Bregma) (Figure S1 A). As reported previously [22], a single cortical stimulus evokes negligible dorsal root potentials, and a repetitive stimulation is required to produce large responses. Control C–fiber EFPs evoked by SN stimulation were compared with those that occurred 25 to 100 ms after cortex stimuli. Due to the variability in the location of stimulating and recording electrodes in each experiment, the time interval between SN stimulation and cortex stimulation was adjusted in each experiment.

Using the same protocol, we tested the effects of the GABA_A_ receptor antagonist BIC (20 µM dissolved in the saline solution irrigating the surface of the spinal cord) on the inhibition of C–fiber EFPs produced by sensorimotor cortex stimulation. The cortex-mediated inhibition of C–fiber EFPs was tested before, every 10 min during BIC administration, and after the wash.

### Post tetanic potentiation of C–fiber evoked responses

C–fiber EFPs were evoked by single constant current pulses delivered every 10 s. Following a 15–min baseline period, a high frequency stimulation (HFS) was delivered in one (100–ms or 250–ms) train (pulse duration 0.1 ms, 100 Hz, 100 µA). Recordings of the C–fiber EFPs then continued for 1 h post–tetanus. In order to analyze the cortical stimulation effects, the time course of post tetanic potentiation (PTP) produced by SN alone was compared with the PTP produced by the tetanic stimulation of SN delivered together with a cortical stimulation train. Cortical stimulation preceded the HFS to the SN by 50 ms.

### Paired pulse test

To determine the influence of the sensorimotor cortex on paired pulse depression or facilitation of C-fiber EFPs, two consecutive SN stimuli were applied, separated by a 200-ms interval. In this way, BIC (100 µM, 50 µl) was applied onto the surface of the sensorimotor cortex. Changes in the ongoing cortical and dorsal horn activity evoked by cortical BIC administration were correlated with changes in the PPR C-fiber responses evoked by sciatic nerve stimulation.

### Measurement of excitability of terminal arborizations

These experiments were conducted according to the method developed by Wall [30] and described in the rat later on [26], [31]. Briefly, the SU nerve was mounted on silver hook electrodes for recording compound antidromic responses. The antidromic responses were produced by stimulating the afferent terminals by means of coated tungsten micro–electrodes located in L4–L5 at the entry zone of dorsal roots in the superficial dorsal horn. The stimulation electrodes were located at the site where maximal antidromic responses in the SU nerve were recorded (300–400 µm deep), corresponding to the negative focus of SU nerve EFPs (Figure S2 C). Stimulus strengths (<500 µA; 100 µs; 0.7 Hz) were adjusted until a stable compound action potential was recorded on the SU nerve; the stimulation strength was always submaximal. The conduction velocity and activation threshold of this compound action potential were in the C–fiber range. Conduction velocities (1.88±0.36 m/s) were computed using the latency shifts and the distance between the two recording electrodes located in the SU nerve (Figure S2 A). Additionally, to further demonstrate that the fibers involved had a high activation threshold, a collision test was performed. In this way, the stimulus intensity necessary to evoke a collision of the slow antidromic compound action potential produced by dorsal horn stimulation, exceeded the threshold of the most excitable fibers in the nerve by 30 fold (Figure S2 B). When primary afferent axons were depolarized, they became more excitable, and fixed–intensity stimulation activates more axons; thus, the compound antidromic action potential increases. The size of the antidromic compound action potential evoked by dorsal horn stimulation was evaluated at different intervals preceding cortical stimulation.

### Data analysis

All the recorded EFPs were averaged (16–24 sweeps) and analyzed off line with the Clamfit software (pCLAMP 10.0, Molecular Devices). Statistical analyses were computed using non-parametric tests. The amplitudes of the control C–fiber EFPs, preceded or not by cortical stimulation, were compared using a Wilcoxon test. For multiple comparisons a Kruskall-Wallis ANOVA and Friedman test were performed. Differences were considered significant starting at p<0.05.

## Results

Intraspinal EFPs produced by SU and SN stimulation with long latencies (80–150 ms) were analyzed. In order to better estimate the conduction velocity of the fibers producing C–fiber EFPs, the responses produced by two separate stimulating electrodes were compared to calculate the latency difference in 5 experiments. The conduction velocity estimated in this way corresponds to the activation of C-fibers (1.3±0.2 m/s) (Figure S3 A). Additionally, the electrodes were situated 300–400 µm deep, where the maximal amplitude responses were recorded (Figure S3 C) [32]. We assumed that EFPs produced by SN stimulation are due to C-fiber activation based on their long latencies (80–150 ms), high thresholds (0.3–0.6 mA with 0.1-ms pulse duration) and maximal negativity in the superficial dorsal horn [6], [32], [33], [34]. In each experiment, the SN stimulation strength was adjusted to produce a C–fiber EFP between 40 and 60% of the maximal response (0.5–0.8 mA) (Figure S2 B).

### Sensorimotor cortex stimulation inhibits dorsal horn C-fiber EFPs

**Figure 1:**
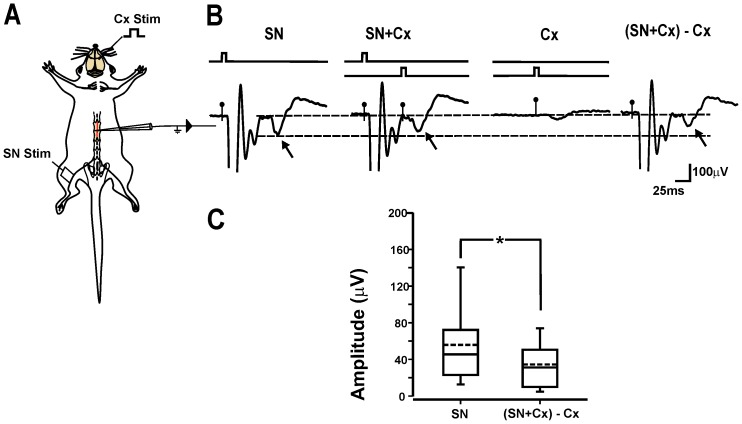
Sensorimotor cortex stimulation inhibits C–fiber dorsal horn EFPs. A, Schematic diagram of the experimental arrangement. B, electrophysiological recordings show: the averaged (n = 16 sweeps) C–fiber EFPs (arrow) generated by stimulation of the sciatic nerve (SN) (first trace); averaged C–fiber EFP evoked by an SN stimulus preceded by cortical stimulation (Cx+SN) (second trace); Cortex–evoked potentials (Cx) (third trace); averaged C–fiber EFP preceded by cortical stimulation minus cortex–evoked potentials ((Cx+SN)–Cx) (fourth trace). C, box plot computed from the amplitudes of C–fiber EFPs evoked by SN stimulation alone and when C–fiber EFPs were preceded (25 to 100 ms) by cortical stimulation ((Cx+SN)–Cx). *p<0.05, Wilcoxon test.

As a first step, the effect of cortical stimulation on C–fiber EFPs was analyzed. Thus, the control nociceptive responses alone were compared with the C–fiber EFPs preceded by cortical stimulation. We observe a significant inhibition of C-fiber EFPs. However, as reported previously, the magnitude of the inhibition depended on the time interval between cortical stimulation and C–fiber EFP [6]. Here, the significant inhibition effects (39.2±4.2% with respect to control; p = 0.0003, Wilcoxon test; n = 17) were obtained when cortical stimulation preceded C–fiber EFPs with time intervals between 25 to 100 ms (Figure 1).

### Effects of sensorimotor cortex stimulation on PTP of C–fiber EFPs

To analyze the mechanisms by which sensorimotor cortex stimulation inhibits C–fiber EFPs, we first tested whether cortical stimulation modulated the time course of PTP of C–fiber EFPs. PTP principally occurs due to an increase in the release probability in response to an action potential resulting, in large part, from the buildup of calcium in the presynaptic terminals during the stimulus trains [28]. In order to exclude the possibility that postsynaptic mechanisms are involved in the augmentation of the amplitude observed following tetanical stimulation, the effects of an NMDA receptor antagonist CPP ((2-carboxypiperazin-4-yl)propyl-1-phosphonic acid; 10 µM, Sigma) were tested. No statistical differences were observed in the PTP produced by a 100-ms stimulating train in the presence of CPP (Figure S4 B), suggesting that postsynaptic NMDA receptors are not involved in the augmentation of the C-fiber EFPs.

**Figure 2:**
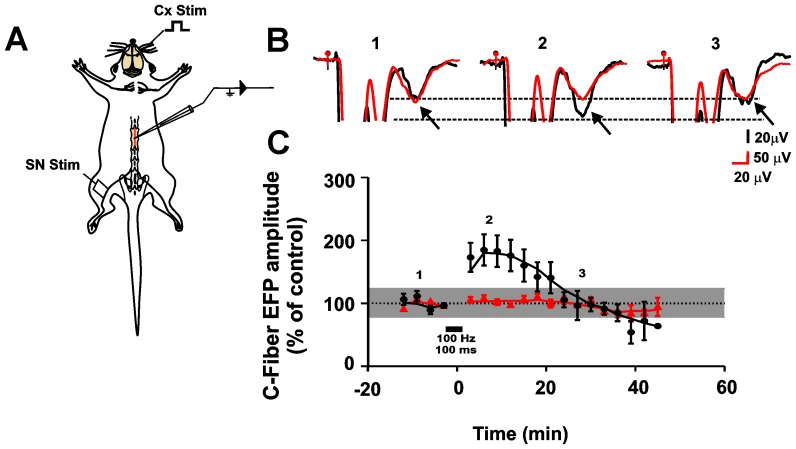
Effect of sensorimotor cortex stimulation on PTP of C–fiber EFPs. A, Schematic diagram of the experimental arrangement. B, averaged (n = 16) electrophysiological recordings obtained before (1), after 10 min (2), and after 20 min (3) of high-frequency stimulation of the SN in control conditions (black) and when the SN high-frequency stimulation was applied in parallel with high-frequency stimulation of the contralateral sensorimotor cortex (red). **C**, PTP temporal course of C–fiber EFPs after a stimulation train of 100 ms delivered to the SN alone (black symbols) and when the SN high-frequency stimulation was applied in parallel with high-frequency stimulation to the contralateral sensorimotor cortex (red symbols). The symbols outside the gray band are statistically different from the basal responses (Friedman test, p<0.05).

When a 100-ms, high-frequency (100 Hz) stimulation (HFS) train was applied to the SN, the amplitude of the C-fiber EFPs increased significantly (Figure 2), reaching a maximum 10 min after the train (182.9±25.1% with respect to control values; p<0.0001, Friedman Test; n = 6). Gradually, the amplitude of the C–fiber EFPs returned to basal levels and 20 min after HFS of the SN, there was no statistical difference with respect to pre-stimulation amplitudes.

However, when the HFS to the SN was applied simultaneously with HFS to the contralateral sensorimotor cortex (pulse duration 0.1 ms, 100 Hz, 100–300 µA), the amplitude of the C–fiber EFPs did not increase when compared to the control (p = 0.25, Friedman test, n = 6) (Figure 2). In addition, cortical stimulation significantly reduced the duration of PTP produced by a longer stimulation train (250 ms) (Figure S4 A).

### Effects of sensorimotor cortex activation on PPR of C–fiber EFPs

**Figure 3:**
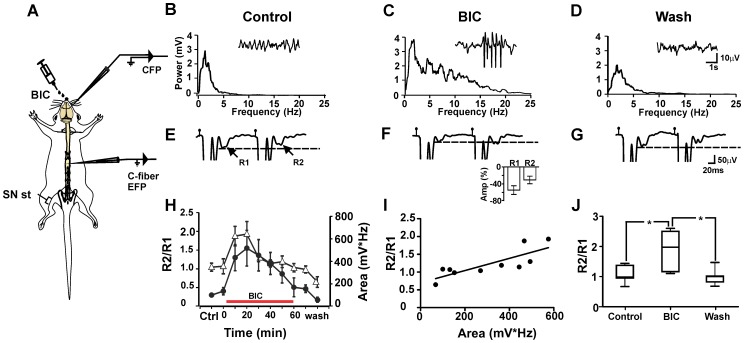
Sensorimotor cortex modulates the PPR of C–fiber EFPs. A, Schematic diagram of the experimental arrangement. B, Power spectrum of the spontaneous cortical activity recorded for 30 sec. The insert shows a sample of spontaneous field potential recorded in the sensorimotor cortex (1000 µm depth, 2.5 mm caudal to bregma, and 3 mm from the midline). C, the same but after 20 min of BIC topical administration (100 µL, 100 µM) on the surface of the sensorimotor cortex. D, the same but after wash. E–G, representative electrophysiological recordings (arrows) of C–fiber evoked responses (R1 and R2) produced by a pair of consecutive stimuli in the SN in control conditions (E), after BIC administration (F), and during wash (G). The insert shows the percent inhibition of the C–fiber EFPs R1 and R2 after 20 min of BIC administration onto the cortex. H, Changes in the PPR (R2/R1) (triangles), and the area below the curve computed from the power spectra of cortical activity during BIC administration (circles). **I**, graph showing a significant positive correlation between the PPR and the area below the curve computed from the cortical activity power spectra (r = 0.86). **J**, box plot of the PPR computed in 7 experiments in control conditions, after 20 min of BIC administration, and during wash. *p<0.05, Kruskall–Wallis ANOVA.

We analyzed the changes in the PPR during different levels of background activity in the sensorimotor cortex. After a control period, BIC (100 µM) was administered on the surface of the sensorimotor cortex (Figure 3A). The PPR as well as the cortical and spinal cord background activities were tested every 10 min. We observed a gradual increase in the area under the curve of the power spectrum computed from cortical and spinal cord activities, reaching a maximum 20 min after starting BIC administration, then gradually decreasing, and returning to basal levels 60 min after BIC withdrawal (Figure S5 A–H). A significant correlation (Spearman r = 0.6, p = 0.03) was observed between the cortical and spinal cord activities, following BIC administration in the sensorimotor cortex (Figure S5 I). During this time, the amplitude and PPR of the C–fiber EFP were tested. The amplitude of C–fiber EFPs decreased after 20 min of BIC cortical administration (54.4±10.1%; p = 0.01; Kruskal–Wallis test; n = 7) along with by an increase in the PPR (Figure 3J). Similar time courses were observed for the PPR and for the area under the curve computed from the cortical activity power spectrum before and after BIC cortical administration (Figure 3H). Moreover, a significant, positive correlation (Spearman r = 0.86; p = 0.002) between the PPR and the power spectrum was observed (Figure 3I), suggesting a presynaptic modulation of C primary afferents that is mediated by the sensorimotor cortex. The administration of vehicle (saline solution) did not change the cortical activity (data not shown). The amplitude of the C–fiber evoked responses was reduced during the period of increased cortical activity.

### Sensorimotor cortex modulates intraspinal excitability of C afferent terminals

**Figure 4:**
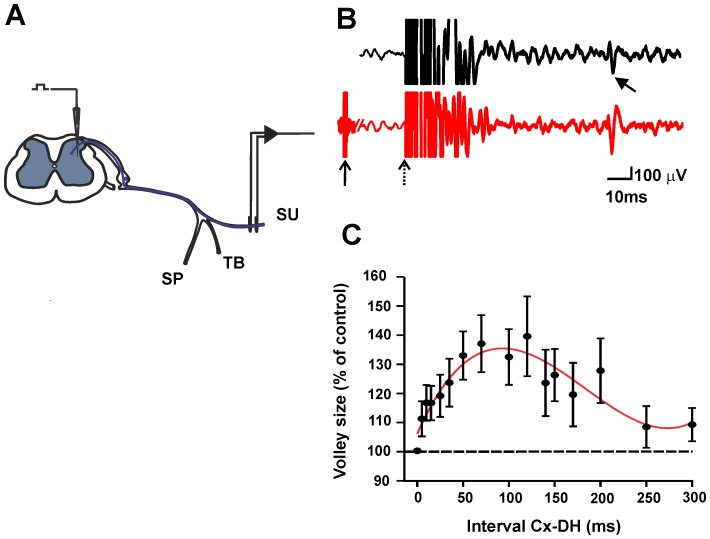
Time course of changes in excitability of slow conducting primary afferent terminals after stimulation of the contralateral sensorimotor cortex. A, experimental arrangement. B, antidromic slow conducting compound action potentials (bold arrow), recorded in the SU nerve, produced by superficial L4 dorsal horn stimulation alone (black) and when the this stimulation was preceded by sensorimotor cortex stimulation 70 ms earlier (red). Arrows indicates the dorsal horn (dotted arrow) and cortex (solid arrow) stimulation times. C, graph shows the amplitude of antidromic volleys at various intervals after the cortical stimulation. Amplitudes are expressed as percent of the amplitude of control antidromic action potentials. SU, Sural nerve; TB, Tibial nerve; SP, Peroneal nerve.

To investigate whether the corticospinal projection modulates the excitability of afferent terminal arbors of C primary afferents, we analyzed the effects of cortical stimulation on the slow antidromic SU compound action potentials evoked by dorsal horn stimulation (threshold 320±54 µA), and recorded in the SU nerve. The excitability of the C afferent terminals, inferred from the height of the compound action potential (conduction velocities less than 2 m/s), was then calculated at different time intervals between cortical and dorsal horn stimulation (Figure 4A–B). We observed a significant increase in the amplitude of the slow antidromic action potential at time intervals between 70 and 120 ms (p<0.0003; Kruskal–Wallis test; n = 12). The time course of the changes in excitability is similar to that of the dorsal root potentials (DRP) evoked by cortical stimulation.

### GABA_A_ receptors are involved in the corticospinal modulation of C-fiber EFPs

**Figure 5:**
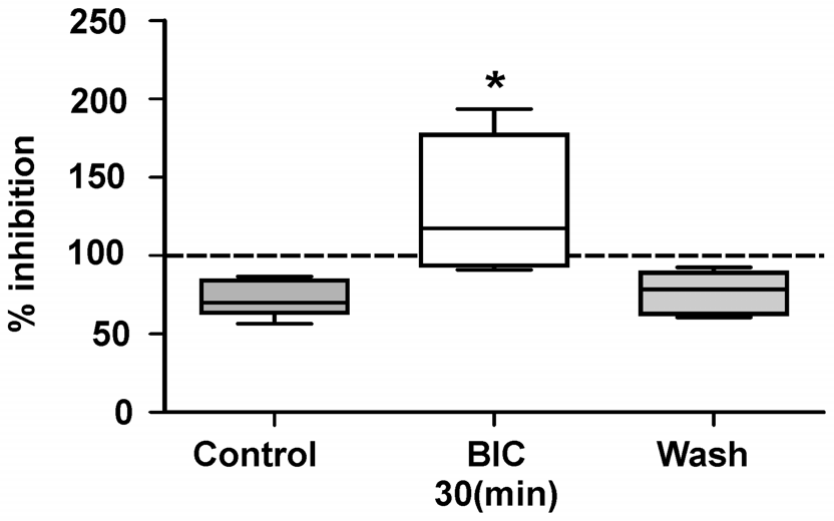
GABAA receptors mediate the cortical inhibition of C–fiber EFPs. Box plot of the inhibition of C–fiber EFPs produced by cortical stimulation before (control), 20 min after BIC administration on the surface of spinal cord, and after wash. Significant inhibition of the amplitudes of C–fiber EFPs produced by cortical stimulation, compared to controls without cortical stimulation (100%) is indicated in the gray bars (p<0.05; Wilcoxon test). Asterisk indicates significant differences with respect to the control (p<0.05; Kruskal-Wallis test). Notice that the cortical evoked inhibition is suppressed by BIC.

To analyze if GABA_A_ receptor activation is involved in the modulation of C–fiber EFPs mediated by the sensorimotor cortex, the effect of intrathecal BIC was evaluated in 6 experiments. The inhibition of C-fiber EFP amplitude produced by sensorimotor cortex electrical stimulation (27.9±4.5% with respect to control) was significantly blocked after 30 min of BIC administration (p = 0.001, Friedman test, n = 6). Moreover, 60 min after washing, the block of the inhibition in C–fiber responses produced by cortical stimulation was completely reversed (Figure 5). Additionally, the amplitude of the C–fiber evoked responses did not change significantly before (42.7±5.18 µV) or during (39.3±6.0 µV) BIC administration (p = 1.0; Wilcoxon test; n = 6), suggesting that these effects are not due to a GABAergic tonic disinhibition.

### Sensorimotor cortex activates dorsal horn interneurons receiving synaptic input from fast conducting cutaneous afferents

**Figure 6:**
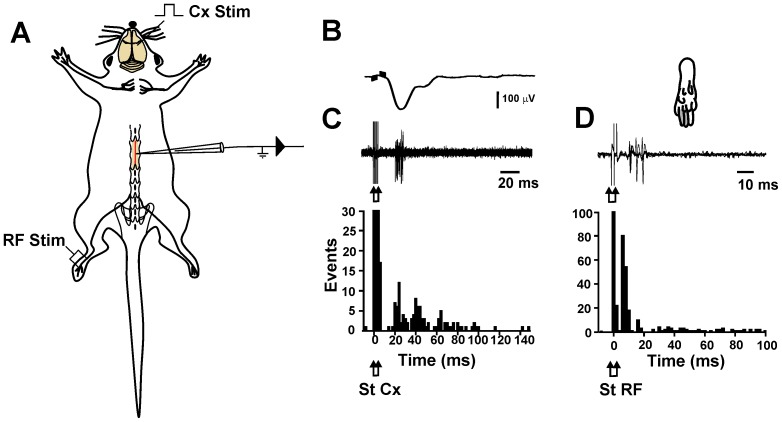
Dorsal horn neurons responding to sensorimotor cortex stimulation also receives input from larger cutaneous afferent fibers. A, experimental arrangement. B, averaged cortical EFP recorded at a depth of 300 µm (top trace). C, extracellular recording of a single neuron in the same experiment (bottom trace) and peri-stimulus time histogram computed from the action potential responses evoked by cortical stimulation of 10 recorded neurons. D, the trace shows superimposed action potential responses evoked by electric stimulation of the receptive field (RF) (represented in the paw drawing) of the same neuron recorded in C, and peri-stimulus time histogram computed from the action potential responses evoked by the RF stimulation of the same recorded neurons that respond to cortical stimulation. The arrows indicate the cortical (Cx) and RF stimulation time.

**Figure 7:**
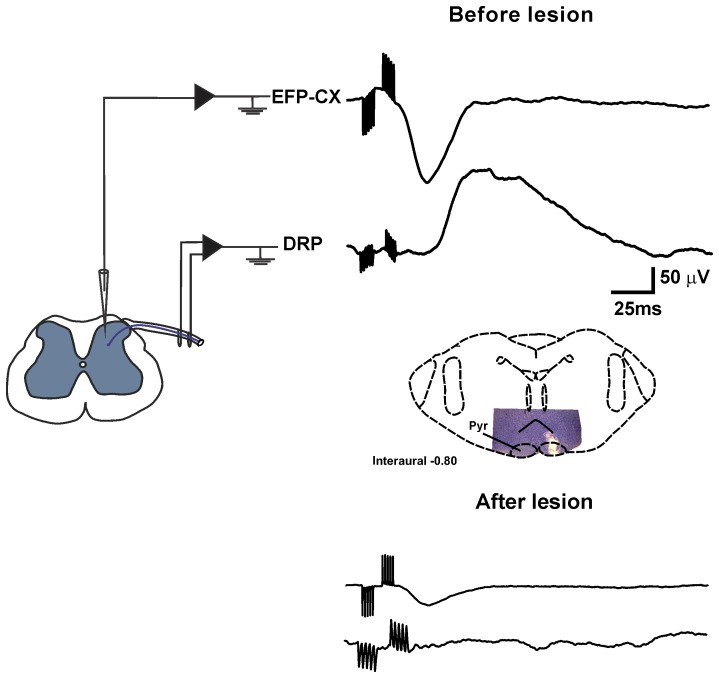
Cortical EFPs and DRPs are suppressed after pyramidal tract lesions. Averaged EFPs (top traces) and DRPs (bottom traces), evoked by contralateral sensorimotor cortex stimulation, recorded in the L4 spinal cord segment before and after electrolytic lesion of the ipsilateral pyramidal tract at the medullary level. The schematic drawing shows the lesion produced in the pyramidal tract. Notice that both DRPs and EFPs are suppressed after pyramidal tract lesion.

Next we investigated the location of the neurons in the spinal cord that are activated by the sensorimotor cortex. In 6 experiments, intraspinal recordings were made at various depths in four parallel tracks separated by 300 µm. Intraspinal EFPs produced by contralateral sensorimotor cortex stimulation were recorded. These cortical EFPs were negative in the superficial dorsal horn, acquired their maximal amplitude between 200 and 400 µm, and their polarity reversed in more ventral regions of the spinal cord (Figure S1 C–D). Moreover, the extracellular unitary activity of the neurons that were activated by sensorimotor cortex stimulation demonstrated that action potentials occurred during the maximal negativity of cortical EFPs (n = 10) (Figure 6A–C). All recorded neurons also responded to cutaneous mechanical and electrical stimulation. The receptive fields (RF) were small and when activated by electric stimulation had low activation thresholds (10–100 µA). The latency of activation after cutaneous electrical stimulation (6.8±0.8 ms) indicates that these neurons also receive information from Aβ fibers (Figure 6D). Similarly, cortical stimulation produced DRPs that started just after the maximal negativity of the cortical EFPs (Figure 7). After an electrolytic lesion of the pyramidal tract (100 µA, 10 s), just before pyramidal decussation, there was a suppression of the cortical EFPs, as well as of the DRPs (Figure 7), suggesting that the dorsal horn neurons were modulated directly by the corticospinal projection.

## Discussion

In the present work we show that after sensorimotor cortex stimulation, the evoked C–fiber spinal field potentianls are inhibited. Although the inhibition of nociceptive neuronal responses mediated by sensory and motor cortices has been previously reported in animal models [4], [5], [6], this paper describes for the first time the possible mechanisms mediating the corticospinal modulation of synaptic dorsal horn responses elicited by slow conducting primary afferents. In order to determine if the sensorimotor cortex modulates the C–fiber neuronal responses at a presynaptic level, we first analyzed the effects of cortical stimulation on PTP as well as on the PPR of C–fiber EFPs. We show that cortical stimulation prevents generation of the PTP and increases the PPR of C–fiber EFPs. These results suggest that the cortical stimulation decreases the probability of neurotransmitter release from slow-conducting afferents [28]. Also, we used Wall's method [30] to analyze whether this effect is due to a change in the excitability of terminal arborizations, and hence a PAD of nociceptive afferents. Using this method we assessed if slow conducting antidromic potentials in the SU cutaneous nerve are modulated by cortical stimulation. We found that sensorimotor cortex activation increases the excitability of nociceptive afferents with a time course similar to that of the DRPs produced by cortex stimulation, suggesting that the EFP inhibition mediated by sensorimotor cortex stimulation is due to a presynaptic control of slow conduction afferents.

In agreement with our results, previous studies have demonstrated that long-latency (80–150 ms) potentials evoked by sural [32] and sciatic [34] nerve electrical stimulation with maximal negativity in the superficial laminae (200–400 µm below the spinal cord surface) are generated by monosynaptic activation of C afferents. Because the second order synapses in the dorsal horn drives a heterogeneous population of neurons [35], some of them excitatory and others inhibitory, a polysynaptic pathway would not be linear. However, there is a direct relationship between the magnitudes of intraspinal field potentials in lamina II and C-fiber nerve volleys. This finding strongly suggests that these intraspinal field potentials are generated by the synapses between the C afferent fibers and the second–order neurons. In contrast, a non–linear relationship was found between the intraspinal field potentials recorded deep in lamina V and C-fiber nerve volleys, indicating a polysynaptic C-fiber input to lamina V neurons [32]. Here, we only analyzed the intraspinal evoked potentials recorded at depths between 300 and 400 µm, corresponding to lamina II [6]. Our data suggest that the intraspinal components recorded are generated monosynaptically.

PTP is a short–term form of plasticity that is reflected in an increase of synaptic transmission produced by a presynaptic stimulation train. This phenomenon is accompanied by an increased probability of release that may result from increased calcium entry into presynaptic terminals [36]. This implies that PTP is principally due to a change in the neurotransmitter-release machinery itself [28], [29]. Despite the fact that PTP is generally thought to be entirely presynaptic, a postsynaptic component has also been suggested [37]. PTP in the spinal cord has been explored in synapses between low–threshold primary afferents and spinal cord neurons [38], [39]. Here we analyzed PTP responses mediated by slow-conducting afferents. In particular, the synapses between C–fibers and second order neurons are plastic and exhibit NMDA receptors dependent long term potentiation. To determine whether postsynaptic NMDA receptors contribute to the PTP seen here, we tested the effect of the NMDA receptor antagonist CPP. We found that CPP did not affect PTP produced by a 100-ms stimulation train, suggesting that postsynaptic mechanisms are not involved in this particular potentiation.

The time course of PTP depends on the stimulation train duration and stimulus frequency [29]. Here, we observed that after a 100–ms high frequency stimulation train, there is an increase of synaptic nociceptive responses that lasts for 20 min. Interestingly, we found that cortical stimulation suppressed the augmentation of nociceptive responses produced after a high-frequency stimulation. Moreover, the duration of PTP produced by a longer stimulation train (250 ms), was reduced. Diverse mechanisms could explain how cortical stimulation abolishes PTP of C-fiber EFPs. However, since PTP is principally due to a change in the presynaptic machinery for neurotransmitter release, we can presume that somatosensory cortex inhibits neurotransmitter release from primary C afferents because of a GABA_A_ PAD and presynaptic inhibition. If so, the same effects should be obtained producing PAD by other means, for example by stimulating other cutaneous afferents or other supraspinal structures.

Another form of short–term synaptic plasticity is the paired–pulse facilitation and depression. These forms of plasticity result from changes in the release probability, which depends on the recent history of synapse activation [28]. Changes in the PPR are an indication to assess changes in neurotransmitter release probability in patch clamp as well as in *in vivo* field potential recordings [40]. In the present study, the PPR was analyzed during different states of ongoing activity recorded in the sensorimotor cortex. To this end, BIC was administered at the surface of the sensorimotor cortex to modify the background electrical activity of the cortex. In this way, the cortical activity, measured as the area under the curve of the power spectrum, increased significantly 20 min after BIC cortical administration. In addition, the PPR of these responses increased, indicating a decrease in the probability of neurotransmitter release. Following BIC administration in the sensorimotor, not only was there an increase in the cortical activity, but also in ongoing spinal cord activity, suggesting that corticospinal neurons were activated by BIC. Previously [6], we have shown that when ongoing cortical activity is suppressed by cortical spreading depression, C–fiber EFPs are increased. Taken together, our results indicate that the sensorimotor cortex exerts a tonic control of nociceptive responses at the dorsal horn level. Moreover, it is possible that any change in the ongoing cortical activity modifies the release probability of the slow conducting afferents that have an impact on nociceptive information. Spontaneous or background cortical activity correlates with the behavioral state [41]; hence, pain perception might depend on behavioral condition. Accordingly, the behavioral state (grooming, alertness, resting, light sleep, deep sleep) was recently reported to have a dramatic effect on pain sensitivity [42].

The effect of cortical activity on PTP and the PPR of C–fiber EFPs suggested that the corticospinal modulation of nociceptive neuronal responses might be exerted at a presynaptic level. Presynaptic control of dorsal horn noxious neuronal responses produced by sensory and motor cortex stimulation has been proposed [2], [3], [4], [5] and the actual set of data are consistent with the presynaptic hypothesis. A well-studied mechanism of presynaptic inhibition in the spinal cord is PAD [43]. In particular, presynaptic inhibition of nociceptive information mediated by PAD, measured as an increase in the excitability of C afferent fibers, has been reported previously [31], [44], [45], [46]. In fact, sensorimotor cortex stimulation in monkeys and cats produces DRPs [20] as well as presynaptic inhibition [21], [22], [23], [24] in large muscular and cutaneous primary afferents. Here, we measured changes in the excitability of C afferent terminals produced by sensorimotor cortex stimulation using Wall's method [30]; since C-fibers are too small to record intraaxonally, this has never been achieved. The time course of changes in the excitability of C–fibers is similar to that reported previously in the rat [31] and follows the time course of DRP produced by contralateral sensorimotor cortex stimulation. Although DRP produced by cortical stimulation is due to PAD predominantly in large cutaneous and muscular afferents, C-fiber DRPs follows a similar temporal course [46]. Our results suggest that cortical stimulation produces PAD in C–fibers, causing an inhibition of action potential propagation into presynaptic terminals and a concomitant reduction in glutamate release to second order dorsal horn neurons [46]. However, the time course of the inhibition of C-fiber EFP conditioned by cortical stimulation [6] is not equal to the DRP produced by contralateral sensorimotor cortex stimulation. Specifically, there is a significant inhibition also at short intervals (<30 ms), suggesting that other, probably postsynaptic, mechanisms are involved as well.

The latencies of the antidromic compound action potentials (45.4±1.1 ms), recorded in the SU nerve after dorsal horn stimulation, are significant lower (p<0.01; n = 41; Mann-Whitney U) than conduction velocities of orthodromic C-fiber EFP recorded in the dorsal horn (81.2±6.2 ms). This difference could be due to various reasons. First, the orthodromic responses have a different origin than antidromic responses. Orthodromic dorsal horn FPs are evoked by entire nerve stimulation with higher stimulation intensities (0.5–0.8 mA). In contrast, antidromic compound action potentials are evoked by more focal and low intensity (<0.5 mA) stimulation into the dorsal horn. For that reason, it is very likely that only the fastest and most excitable terminals could be activated by dorsal horn stimulation. In any case, the conduction velocities for antidromic (1.88±0.36 m/s) and orthodromic (1.3±0.2 m/s) responses correspond to activation of C-fibers.

The contribution of GABA_A_ receptors in pain processing is well known [47]. However, evidence of the contribution of PAD mediated by GABA_A_ receptors in C–fibers is scarce [43]. Recent evidence has shown that PAD mediated by GABA_A_ receptors residing on axons or terminals of C–fibers has a significant role in spinal pain control [46]. The classical view of the neuronal circuit that mediates PAD and presynaptic inhibition involves, in the spinal cord, a minimal trisynaptic pathway with last–order GABAergic inhibitory interneurons that form axo–axonic synapses with primary afferent fibers [43].However, the experimental evidence for this model is weak, and it is now accepted that presynaptic inhibition is more diverse than previously imagined and has distinct mechanisms for each genetically distinct afferent fiber population [48]. Nevertheless, the fact that BIC completely blocks the inhibition of C–fiber dorsal horn responses produced by sensorimotor cortex stimulation suggests that pyramidal descending fibers activate, either directly or indirectly, GABAergic interneurons that in turn produce PAD in C–primary afferent terminals. Further experiments are necessary to know if different populations of nociceptors are modulated via other mechanisms distinct from GABA-mediated PAD.

Numerous efforts to identify and characterize the PAD–mediating interneurons have been made [49], [50], [51]. In particular, the interneurons that are activated by sensorimotor cortex were also activated by electrical stimulation of cutaneous and muscular afferent nerves and the spike discharge follows the time course of DRP produced in large cutaneous and muscular primary afferent [22]. This suggests that a convergence exists somewhere in the pathways that produce segmentally and descending–evoked DRPs [26]. In accord with these results, here we found that the recorded neurons were activated by sensorimotor cortex stimulation also had a peripheral receptive field and were activated by slight mechanical stimulation. The calculated conduction velocity of the fibers responsible for activating these neurons, correspond to the conduction velocities of cutaneous Aβ fibers reported in the rat [52]. In contrast, we found no neurons related to DRPs. Additionally, the neurons responding to sensorimotor cortex stimulation were distributed between depths of 200 and 400 mm, corresponding to laminae II–III. It remains to be determined if the neurons receiving convergent input from sensorimotor cortex and from low-threshold primary afferents are GABAergic [53] and are responsible for increasing the excitability of slow conducting afferents.

The corticospinal projection constitutes a system with multiple functions that share one characteristic: the cortical modulation of spinal cord activity [1]. One of the most-studied functions of this descending system is motor control, which results from direct and indirect modulation of spinal cord interneurons and motoneurons [54], [55], [56], [57]. This system is also involved in modulating sensory information at the dorsal horn [58], including nociception [6]. In this way, it has been proposed that during the onset of voluntary movements there is a complex selection of sensory information, including muscular, tactile, and nociceptive information, that is required for the proper execution of the movements [59], [60], [61]. However, little is known about the organization of corticospinal projections, the intracortical microcircuitry, and the synaptic interactions in the sensorimotor cortex that may encode cortical output to the spinal cord. Understanding this organization is important in order to know, in an integrative way, the special features of the human motor system that are particularly vulnerable to neurological diseases, including stoke, cerebral palsy, movement disorders, spinal cord injury, and motoneuron disease.

## Conclusion

The sensorimotor cortex modulates nociceptive information carried by C–fibers by means of a presynaptic GABAergic mechanism that involves primary afferent depolarization.

## Supporting Information

Figure S1 **Cortical EFPs.** A, averaged cortical EFPs (top traces), evoked by contralateral sensorimotor cortex stimulation, recorded in the L4 spinal cord segment. The drawing shows the relative position of the stimulation zones in the cortex. The red trace was produced by stimulating a zone located 1.5 mm medially from the blue trace. B, the graph shows the relationship between the stimulation intensity and the cortical EFP amplitude. C, amplitudes of averaged cortical EFPs recorded at different depths from the dorsal surface of the spinal cord computed in 6 experiments. D, isopotential contours of cortical EFP from a series of four parallel recording tracts. Notice that the maximal negativity occurs in the dorsal horn between depths of 200 and 400 µm.
(TIF)

Figure S2 **Antidromic responses recorded in the SU nerve following stimulation of the dorsal horn.** A, conduction velocity of the SU nerve fibers was determined with the upper experimental arrangement. The upper traces show the antidromic responses produced by dorsal horn stimulation (400 µA) recorded at two different locations on the SU nerve and separated by 1.5 cm. B, threshold of the SU nerve fibers producing the late responses was determined with the lower experimental arrangement. The lower traces show the antidromic responses produced by dorsal horn stimulation (solid arrow) accompanied by additional stimulation of the SU nerve (dotted arrow) with strength of 1×T (black trace), 15×T (blue trace) and with strength of 30×T (red trace) of the most excitable fibers. The stimulating electrode in the SU nerve was located 4 mm from the recording electrode. Notice the collision of the antidromic response. The asterisks indicate the point at which the antidromic response would have occurred.
(TIF)

Figure S3 **C-fiber EFPs.** A, averaged C-fiber EFPs (top traces) produced by two stimulating electrodes located 3 cm apart on the sciatic (SN) and sural (SU) nerves. The drawing shows the relative positions of the electrodes. Notice the similarity of the two responses. B, relationship between the stimulation intensity and the amplitude of the C-fiber EFPs. C, relationship between EFP amplitude and recording depth.
(TIF)

Figure S4 **C-fiber EFP PTP does not depend on NMDA receptor activation.** A, PTP time course of C–fiber EFPs after a stimulation train of 250 ms delivered to the SN alone (black symbols) and when the SN high-frequency stimulation was applied in parallel with high-frequency stimulation to the contralateral sensorimotor cortex (red symbols). B, PTP time course of C–fiber EFPs after a 100-ms stimulation train delivered to the SN in control conditions (black symbols) and in the presence of the NMDA receptor antagonist CPP (red symbols). The symbols outside the gray band are statistically different from the basal responses (Friedman test, p<0.05).
(TIF)

Figure S5 **Changes in cortical and dorsal horn ongoing activity following BIC cortical administration.** A, power spectrum of the spontaneous cortical activity recorded for 30 sec. The insert shows a sample of spontaneous field potential recorded in the sensorimotor cortex (1000 µm depth, 2.5 mm caudal to bregma and 3 mm from the midline). B, the same but after 20 min of BIC topical administration (100 µL, 100 µM) on the surface of the sensorimotor cortex. C, the same but after wash. D–F, the same as A–C but power spectra were computed from spontaneous dorsal horn activity recorded simultaneously. G, changes in the area below the curve computed from the power spectra of cortical activity during BIC administration. H, the same as G but for the dorsal horn activity. I, graph showing a significant positive correlation between the area below the curve computed from the cortical and dorsal horn activity power spectra (Spearman r = 0.6, p = 0.03).
(TIF)
