# Supplementary figures and images for: Cortical Presynaptic Control of Dorsal Horn C–Afferents in the Rat
Source: PLoS One. 2013 Jul 30;8(7):e69063. doi: 10.1371/journal.pone.0069063 (PMC3728294; doi:10.1371/journal.pone.0069063)

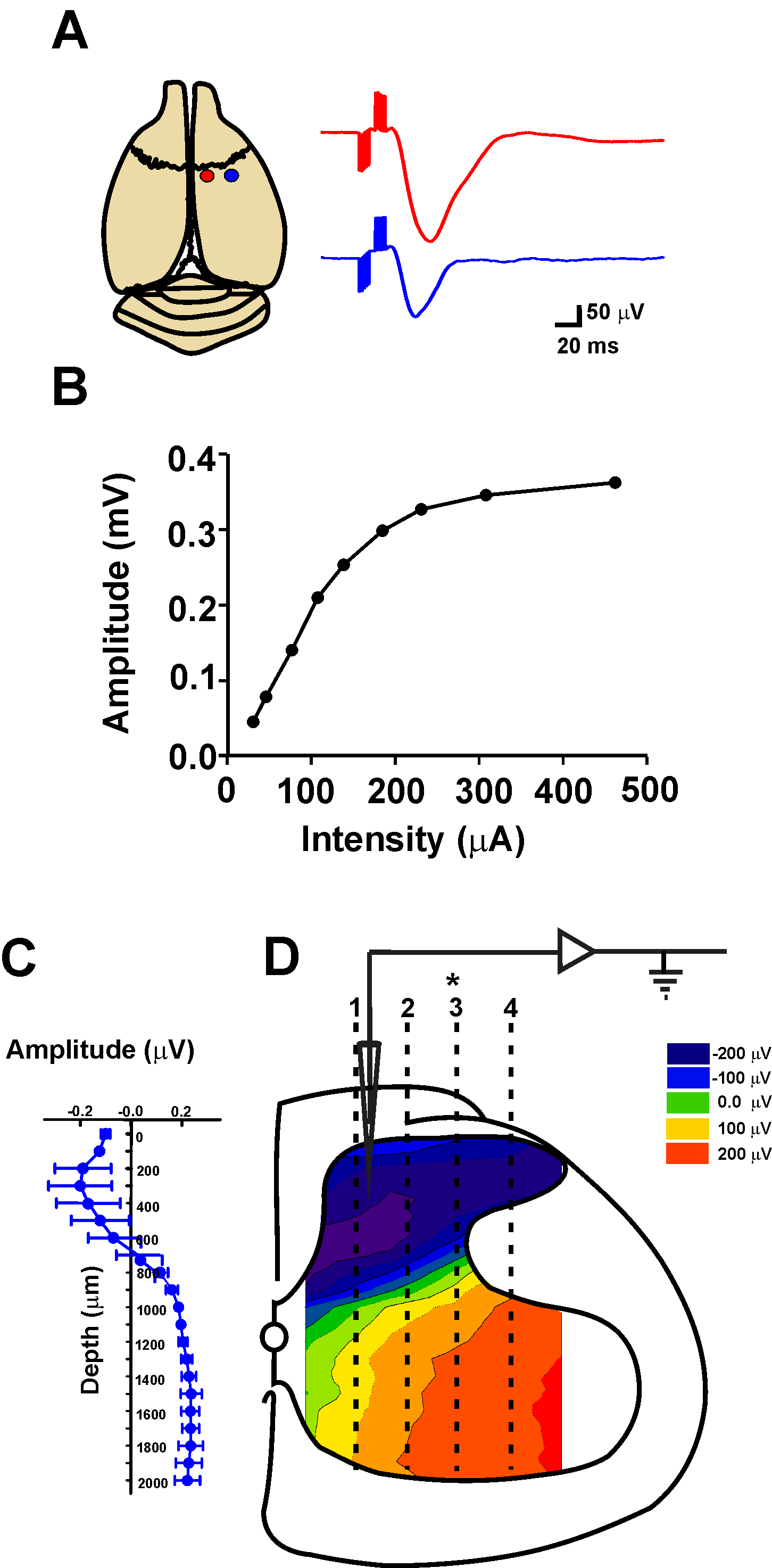

Supplement: Figure S1 — Cortical EFPs. A, averaged cortical EFPs (top traces), evoked by contralateral sensorimotor cortex stimulation, recorded in the L4 spinal cord segment. The drawing shows the relative position of the stimulation zones in the cortex. The red trace was produced by stimulating a zone located 1.5 mm medially from the blue trace. B, the graph shows the relationship between the stimulation intensity and the cortical EFP amplitude. C, amplitudes of averaged cortical EFPs recorded at different depths from the dorsal surface of the spinal cord computed in 6 experiments. D, isopotential contours of cortical EFP from a series of four parallel recording tracts. Notice that the maximal negativity occurs in the dorsal horn between depths of 200 and 400 µm. (TIF) [file pone.0069063.s001.tif]

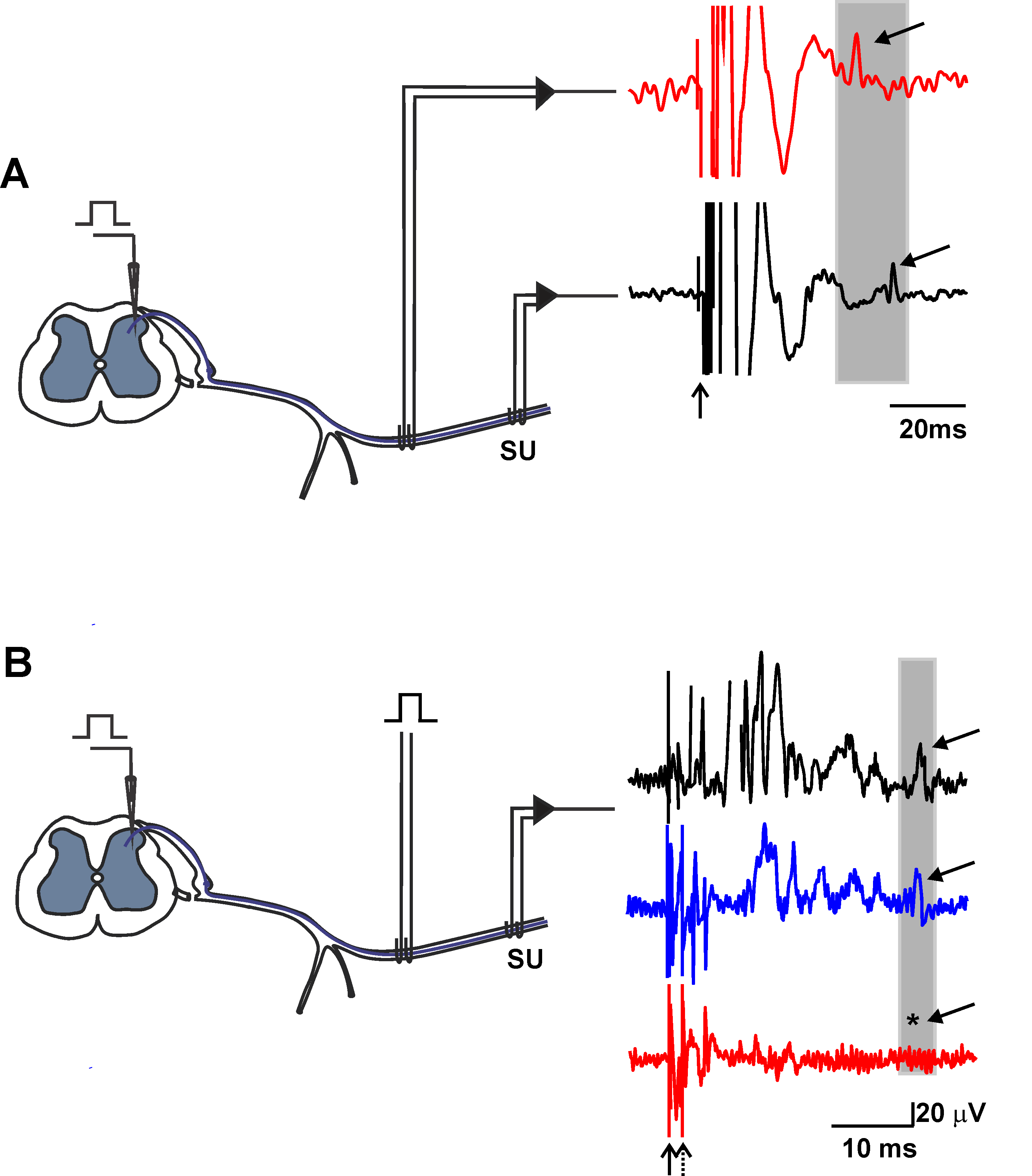

Supplement: Figure S2 — Antidromic responses recorded in the SU nerve following stimulation of the dorsal horn. A, conduction velocity of the SU nerve fibers was determined with the upper experimental arrangement. The upper traces show the antidromic responses produced by dorsal horn stimulation (400 µA) recorded at two different locations on the SU nerve and separated by 1.5 cm. B, threshold of the SU nerve fibers producing the late responses was determined with the lower experimental arrangement. The lower traces show the antidromic responses produced by dorsal horn stimulation (solid arrow) accompanied by additional stimulation of the SU nerve (dotted arrow) with strength of 1×T (black trace), 15×T (blue trace) and with strength of 30×T (red trace) of the most excitable fibers. The stimulating electrode in the SU nerve was located 4 mm from the recording electrode. Notice the collision of the antidromic response. The asterisks indicate the point at which the antidromic response would have occurred. (TIF) [file pone.0069063.s002.tif]

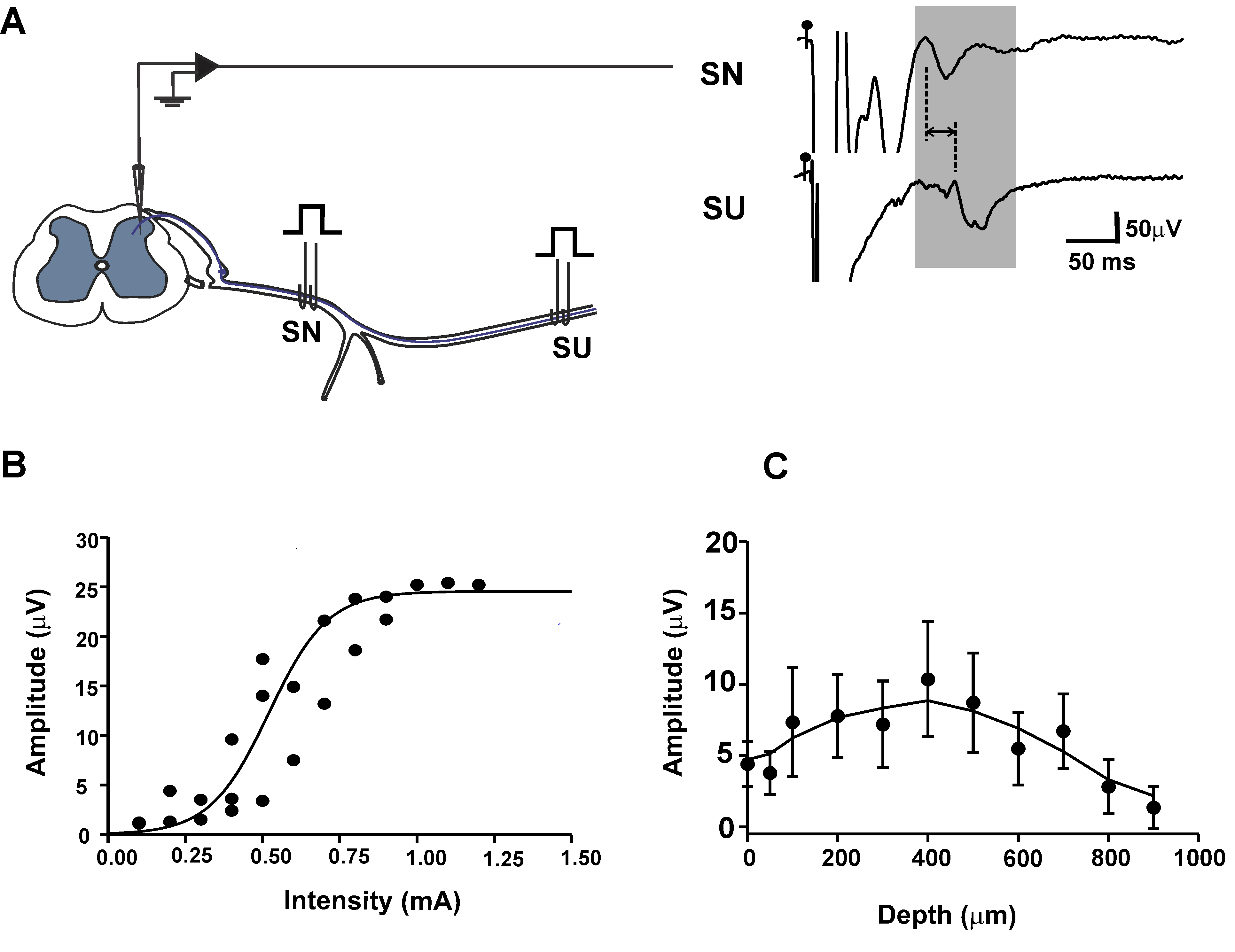

Supplement: Figure S3 — C-fiber EFPs. A, averaged C-fiber EFPs (top traces) produced by two stimulating electrodes located 3 cm apart on the sciatic (SN) and sural (SU) nerves. The drawing shows the relative positions of the electrodes. Notice the similarity of the two responses. B, relationship between the stimulation intensity and the amplitude of the C-fiber EFPs. C, relationship between EFP amplitude and recording depth. (TIF) [file pone.0069063.s003.tif]

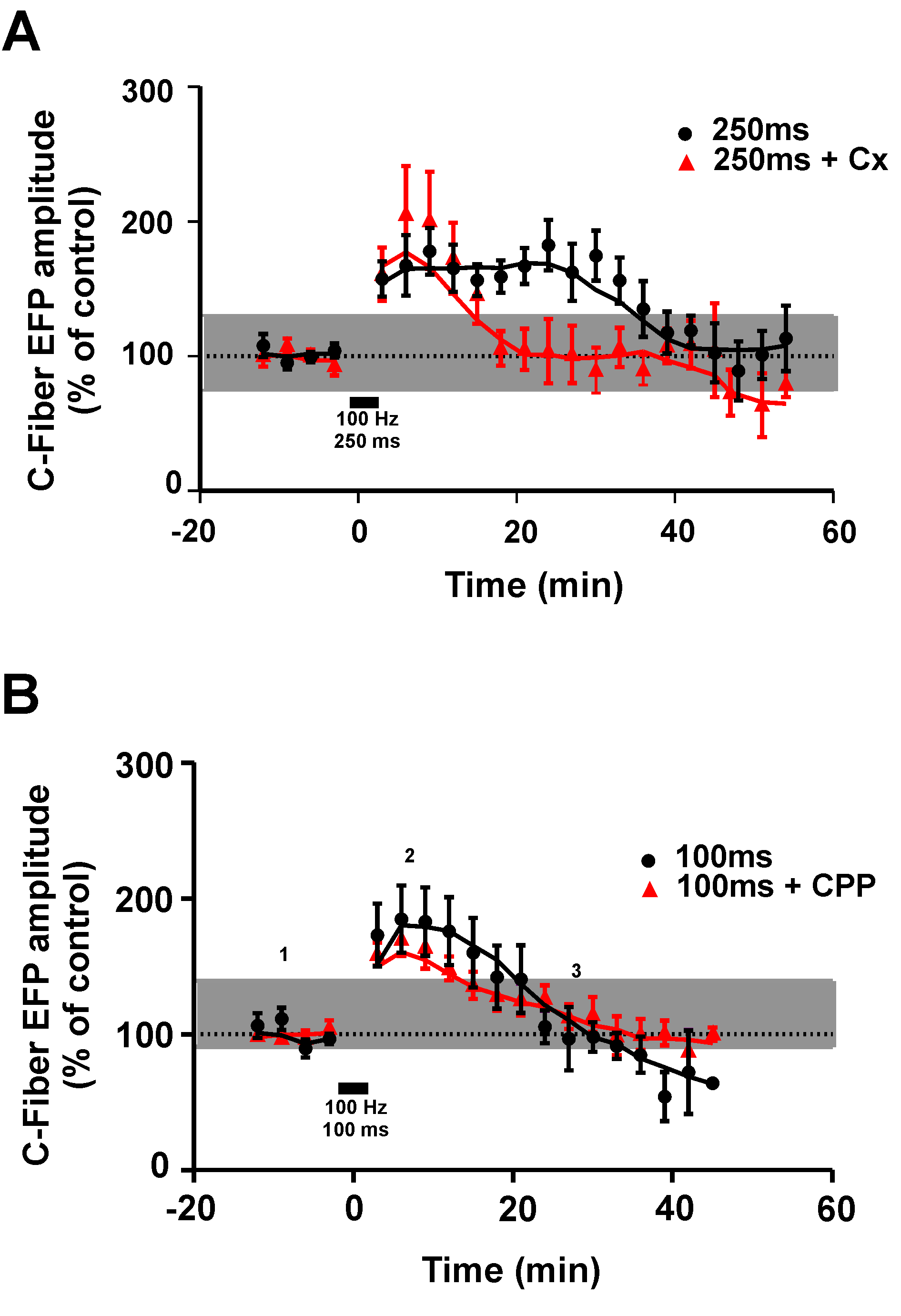

Supplement: Figure S4 — C-fiber EFP PTP does not depend on NMDA receptor activation. A, PTP time course of C–fiber EFPs after a stimulation train of 250 ms delivered to the SN alone (black symbols) and when the SN high-frequency stimulation was applied in parallel with high-frequency stimulation to the contralateral sensorimotor cortex (red symbols). B, PTP time course of C–fiber EFPs after a 100-ms stimulation train delivered to the SN in control conditions (black symbols) and in the presence of the NMDA receptor antagonist CPP (red symbols). The symbols outside the gray band are statistically different from the basal responses (Friedman test, p<0.05). (TIF) [file pone.0069063.s004.tif]

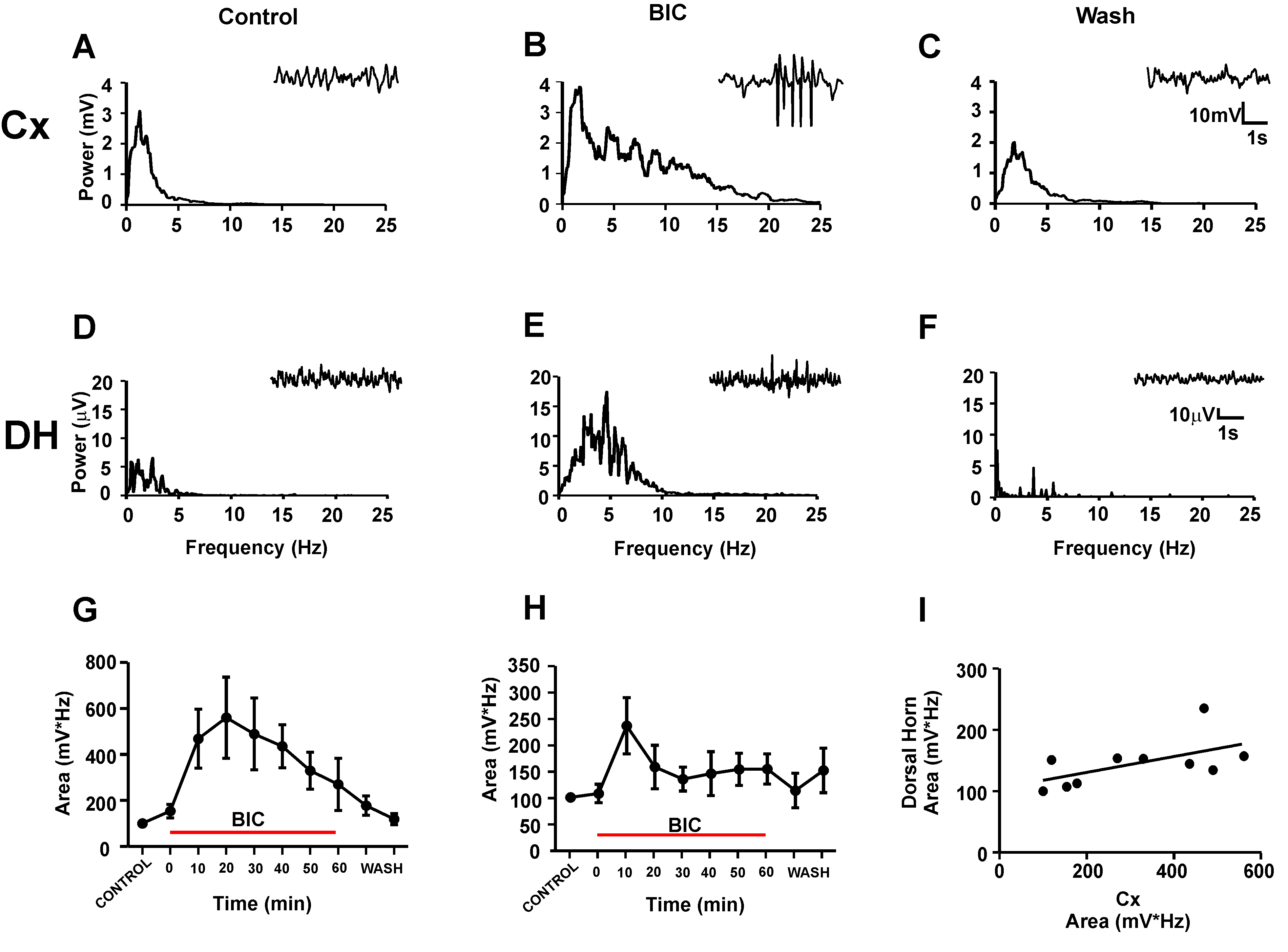

Supplement: Figure S5 — Changes in cortical and dorsal horn ongoing activity following BIC cortical administration. A, power spectrum of the spontaneous cortical activity recorded for 30 sec. The insert shows a sample of spontaneous field potential recorded in the sensorimotor cortex (1000 µm depth, 2.5 mm caudal to bregma and 3 mm from the midline). B, the same but after 20 min of BIC topical administration (100 µL, 100 µM) on the surface of the sensorimotor cortex. C, the same but after wash. D–F, the same as A–C but power spectra were computed from spontaneous dorsal horn activity recorded simultaneously. G, changes in the area below the curve computed from the power spectra of cortical activity during BIC administration. H, the same as G but for the dorsal horn activity. I, graph showing a significant positive correlation between the area below the curve computed from the cortical and dorsal horn activity power spectra (Spearman r = 0.6, p = 0.03). (TIF) [file pone.0069063.s005.tif]
